# Underwater Relative Pose Estimation and Tracking Between AUVs Based on a Hull-Integrated Laser-Beam Marker

**DOI:** 10.3390/s26206576

**Published:** 2026-10-16

**Authors:** Yi Yang, Qingbo Wei, Xingqun Zhou, Zhiqiang Hu, Chuanzhi Fan, Quan Zheng, Zhichao Wang, Dianyou Fu

**Affiliations:** 1 State Key Laboratory of Robotics and Intelligent Systems, Shenyang Institute of Automation, Chinese Academy of Sciences, Shenyang 110016, China; weiqingbo@sia.cn (Q.W.); zhouxingqun@sia.cn (X.Z.); fanchuanzhi@sia.cn (C.F.); zhengquan@sia.cn (Q.Z.); wangzhichao@sia.cn (Z.W.); fudianyou@sia.cn (D.F.); 2 University of Chinese Academy of Sciences, Beijing 100049, China

**Keywords:** autonomous underwater vehicles, relative pose estimation, monocular vision, laser-beam marker, structured feature extraction, closed-loop tracking, underwater optical guidance

## Abstract

Close-range tracking between autonomous underwater vehicles (AUVs) requires continuous six-degree-of-freedom (6-DoF) relative pose feedback, yet target boards and protruding light arrays are difficult to integrate into compact streamlined hulls. This paper presents a monocular relative pose sensing and tracking system based on a hull-integrated laser-beam marker: four blue beams emitted through hull-flush outlets form four calibrated coplanar intersections. A Hough–strip method recovers the principal beams and their intersections from fragmented, merged, and spurious image responses. To suppress the pose jumps that image-point errors induce through the coplanar ambiguity, the proposed solver refines multiple planar candidates and, in each frame, outputs the candidate consistent with both the current observation and the preceding motion. The system runs onboard in real time. In fixed-pose pool tests over 2–6 m, the lateral and attitude errors were at the centimeter and degree levels, respectively, and the normalized optical-axis root-mean-square error (RMSE) was 2.4–2.8%. On dynamic lake data, the proposed solver did not exhibit the large attitude jumps observed for representative perspective-*n*-point (PnP) solvers and reduced the root mean square of the third-order attitude differences to about 1/11 of the single-initialization value. In a 214 s nighttime lake trial, 99.44% of frames yielded valid poses, supporting three-channel closed-loop tracking.

## 1. Introduction

Autonomous relative tracking between underwater robots is fundamental to formation navigation, escort observation, cooperative docking, and autonomous recovery. The follower continuously adjusts its position and attitude according to the leader’s relative motion to maintain a safe separation, a suitable viewing angle, and the desired relative configuration [1,2,3,4,5]. Lateral position, following distance, and yaw provide the most direct feedback for close-range tracking control, whereas depth, pitch, and roll support formation maintenance and operational-state monitoring [6,7,8]. Continuous six-degree-of-freedom (6-DoF) relative pose sensing that can be directly incorporated into the control loop is therefore essential for autonomous underwater tracking.

Because satellite navigation and conventional radio signals are strongly attenuated in water, autonomous underwater vehicles (AUVs) generally fuse information from inertial navigation systems, Doppler velocity logs, pressure sensors, acoustic positioning, and visual observations [9,10,11]. Acoustic systems operate over long ranges and are well suited to remote search, communication, and coarse guidance [12]. In the vehicle-following, close-range tracking, and docking-and-recovery phases, optical observations offer higher spatial resolution and more direct geometric constraints, and are therefore often adopted as the primary short-range feedback for closed-loop tracking [13,14,15]. Most existing underwater optical methods establish image-to-target correspondences using passive target boards or engineered light sources, such as light arrays and optical beacons [7,16,17,18,19,20]. Although these optical targets improve feature detectability, external target surfaces, protruding light mounts, and long physical baselines occupy the limited installation space of compact AUVs, alter their exterior profiles, and increase hydrodynamic drag.

To address these limitations, our previous work used the directions and intersections of multiple line-structured light beams to construct relative guidance observations for underwater robots [21]. Such vector-light patterns can be fully integrated into the vehicle to form a hull-integrated optical marker that provides a monocular camera with geometrically constrained relative pose observations without requiring an external optical target structure. This arrangement can provide 6-DoF relative measurements without altering the exterior profile of a compact AUV. Building on this concept, the present study addresses the observation recovery and pose continuity required for closed-loop autonomous tracking.

Obtaining a stable and continuous pose from a vector-light pattern nevertheless remains challenging on two counts: feature extraction must be reliable under degraded imaging conditions, and the coplanar geometry introduces a pose ambiguity. Underwater scattering, ambient light, suspended particles, and motion blur can fragment beams, merge adjacent responses, and produce spurious detections. The planar geometry formed by the four coplanar intersections is sensitive to image-point perturbations, so candidate solutions with similar reprojection errors may alternate between frames and cause abrupt changes in depth and attitude [22,23,24]. The proposed approach jointly exploits the spatial structure of the vector-light pattern and the temporal continuity of relative motion. Structured line-segment aggregation recovers complete beam observations, while planar-homography initialization, Levenberg–Marquardt (LM) reprojection refinement, and observation-constrained temporal candidate selection suppress branch switching. In each frame, the solver outputs an observation-consistent candidate selected with a motion reference derived from previously accepted poses; it is designed to suppress the pose jumps that underwater image noise induces in dynamic scenes. The resulting relative pose feedback is continuous and suitable for closed-loop control.

**Figure 1:**
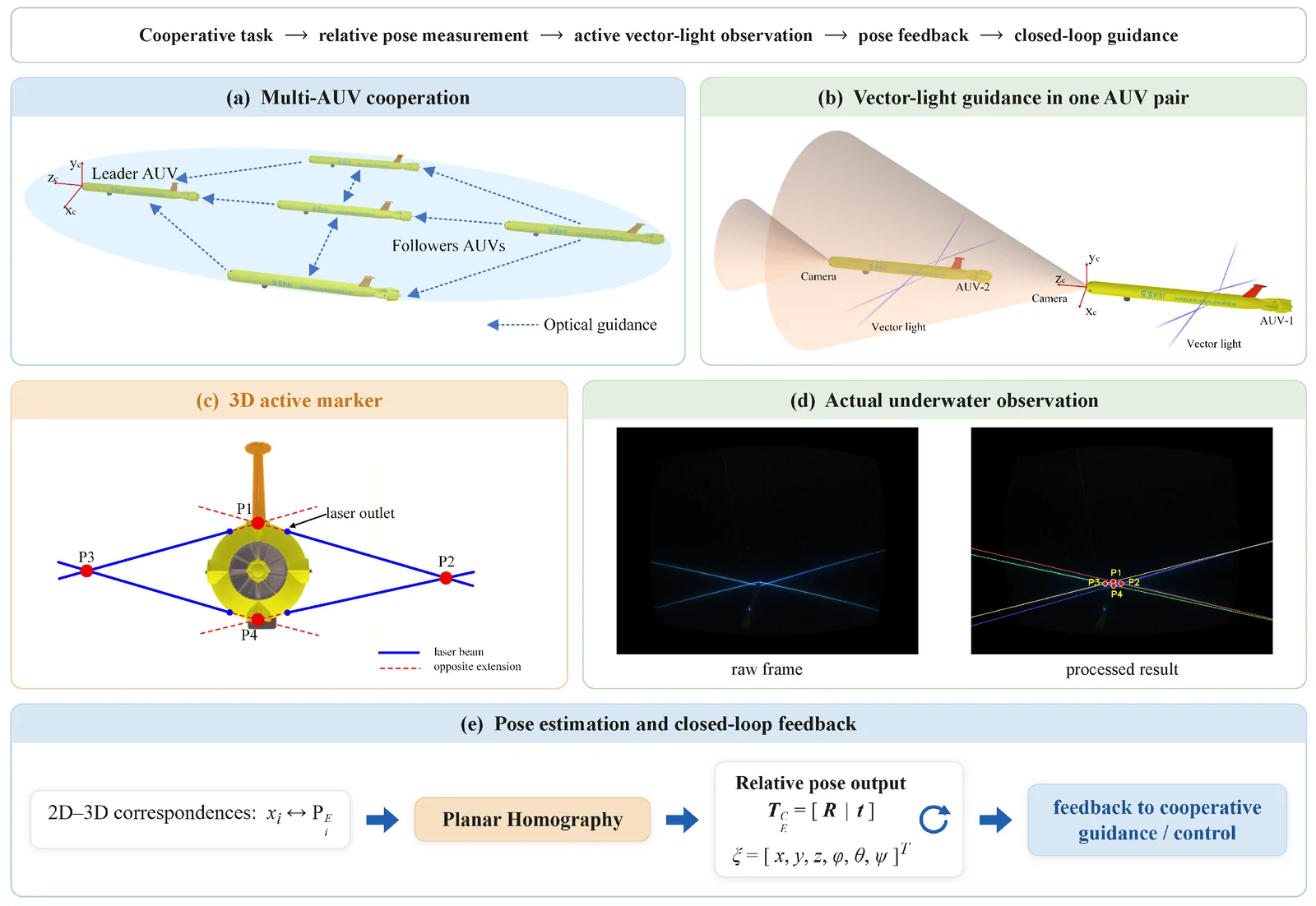
Autonomous underwater robot tracking using vector-light visual relative pose sensing. The upper diagram shows the system pipeline from the tracking task and vector-light observation to relative pose estimation and closed-loop control. (**a**) Underwater robot tracking and cooperation scenario. (**b**) Fore–aft guidance configuration, with the leader ahead of the follower. (**c**) Four collimated beams lie in a common transverse plane perpendicular to the leader’s longitudinal axis and intersect pairwise to form the calibrated feature points $P_{1}$–$P_{4}$. (**d**) A real underwater image and the structured feature extraction result. (**e**) The relative pose $T_{E}^{C}=\left[R|t\right]$ is estimated from the 3D–2D correspondences and fed back to the underwater tracking controller.

The complete relative pose sensing and autonomous tracking system is shown in Figure 1. It integrates active vector-light marking, structured observation recovery, temporally continuous pose estimation, and motion control into a closed-loop pipeline. The main contributions are as follows:

1. A vector-light vision-based system for relative pose sensing and autonomous underwater robot tracking is proposed and implemented. The spatial crossing geometry of the hull-integrated laser-beam marker establishes 3D–2D pose constraints without requiring an external optical target structure, and the system outputs continuous 6-DoF relative poses online, three components of which (lateral position, following distance, and yaw) are fed back to close a three-channel tracking loop.
2. A Hough–strip structured feature extraction method is developed to cope with fragmented, incorrectly connected, and spurious line segments in degraded underwater images. Strip constraints with finite width and gap tolerance aggregate segments from the same physical beam, enabling stable recovery of the four principal beams and their intersections.
3. A pose solver is developed to suppress branch switching caused by the pose ambiguity of four coplanar points. It refines planar candidates from homography, infinitesimal plane-based pose estimation (IPPE), and previous-pose initializations and, through temporally continuous candidate selection (TCS), outputs in each frame the candidate consistent with both the current observation and the preceding motion. Relative to the single-initialization variant, it reduces the root mean square (RMS) of the third-order attitude differences by approximately one order of magnitude while maintaining comparable reprojection errors.
4. Fixed-pose tests against a mechanical reference quantify the accuracy of the sensing system, comparisons on dynamic lake data show how the proposed solver suppresses pose jumps, and a closed-loop lake experiment verifies onboard feedback for three-channel tracking.

## 2. Related Work

### 2.1. Relative Sensing in Multi-AUV Cooperation

Multi-AUV tasks such as formation navigation, vehicle following, and cooperative docking rely on reliable inter-vehicle relative states as closed-loop feedback [1,2,25,26]. Existing approaches can be broadly divided into acoustic and visual methods according to the sensing mechanism used for direct relative measurements. Acoustic methods estimate relative position and constrain dead-reckoning drift through inter-vehicle ranging and navigation-information sharing [27]. Representative implementations include navigation aided by a single maneuvering surface craft [28] and single-beacon one-way-travel-time navigation [29]. Inverted ultra-short-baseline systems further reduce integration costs for compact platforms [30]. These methods operate over long ranges and do not depend on water visibility. Nevertheless, propagation delay, update rate, and angular resolution often limit their outputs to range, bearing, or relative position, making it difficult to directly obtain the full relative attitude required for close-range motion control [31]. Visual methods establish correspondences between image observations and target geometry using the vehicle appearance or engineered optical features. At ranges of several meters, they can estimate relative position or even 6-DoF pose at comparatively high update rates [7,8,18,32]. They have been applied to multi-vehicle following, formation keeping, the close-range stages of docking and recovery, and the detection and tracking of multiple targets in cross-domain operations [13,15,33,34], and can complement acoustic systems by supplementing medium- and long-range guidance with precise short-range feedback. This study focuses on visual relative sensing and seeks to provide continuous 6-DoF relative poses for closed-loop tracking despite limited installation space on compact platforms and degraded underwater imagery.

### 2.2. Underwater Visual Guidance and Optical Markers

Underwater visual guidance commonly uses engineered optical features to establish stable correspondences between image observations and target geometry. Such features can be categorized as passive or active according to whether they emit light. Passive markers include planar coded patterns such as ArUco and AprilTag, as well as three-dimensional geometric markers designed for underwater docking. Known corners or structural features are used to recover the relative pose [19,35,36,37,38]. Image restoration and marker-pattern enhancement have extended the range of beam attenuation coefficients under which these markers remain usable [32]. Although passive markers are well established, they generally require a sufficiently large planar target or three-dimensional structure outside the target AUV or docking station. Their detection range and attitude observability are also sensitive to water turbidity, ambient illumination, and viewing angle.

Active optical markers use light-emitting diode (LED) beacons or laser sources to create visual features with known geometry, improving detectability in weakly textured and low-light environments. Representative systems include LED arrays arranged in circular, L-shaped, or square configurations, as well as docking-guidance devices based on light-source arrays or blue laser diodes [16,17,39,40]. To prevent beacons from leaving the field of view during close approach, a cage-like dual-layer guidance array maintains visual localization through hierarchical matching between its outer and inner light sources [41]. Other studies have improved detection robustness in complex underwater backgrounds using compact optical beacons, modulated sources, or adaptive color strategies [20,42,43]. However, pose constraints in existing systems are still mainly provided by external optical structures. Examples include four high-intensity LEDs mounted on a leader AUV for relative formation positioning [8], four green beacons installed on a docking ring [44], and a triangular coded board combined with tracing lights for 6-DoF pose estimation [45]. For slender, compact AUVs, these target boards, light-array baselines, and protruding mounts occupy scarce hull space and may alter the vehicle profile and hydrodynamic characteristics. Compactly integrating a marker into the hull while preserving explicit pose constraints and underwater detectability therefore remains an important problem in close-range visual guidance.

### 2.3. Pose Recovery from a Small Number of Coplanar Features

Recovering camera pose from 3D–2D correspondences is a classical problem in visual measurement [46,47]. The perspective-*n*-point (PnP) formulation and its efficient variants, such as EPnP [48] and SQPnP [49], are highly practical when numerous feature points are well distributed and image noise is limited, and they are often combined with robust frameworks such as random sample consensus (RANSAC) [50]. For planar or nearly coplanar targets, however, the pose ambiguity is well documented. The reprojection-error function for four coplanar points can contain two similar local minima that become increasingly difficult to distinguish as observation noise grows, causing branch-switching jitter in image sequences [22,23]. Iterative approaches [23,51] mitigate incorrect selection by explicitly searching for and comparing multiple minima, whereas IPPE [24] directly returns candidate solutions using the planar structure. During transitions between near-frontal and oblique views, however, a criterion based only on the current reprojection error may still alternate between branches when their reprojection errors are similar. Other studies render the correct branch observable by adding dedicated patterns to the marker surface [52], which is not feasible for a marker formed by light beams in water. The method proposed here constructs an explicit initialization from the planar homography and selects among multiple pose candidates under both current-observation constraints and temporal continuity. This design suppresses branch jumps with sparse planar observations, as detailed in Section 4.

## 3. Active Vector-Light Marker and Structured Observation

### 3.1. Marker Configuration and Observation Model

In the underwater tracking task shown in Figure 1a, two AUVs form the basic tracking unit (Figure 1b). The leader provides active observation geometry using a vector-light marker, and the follower estimates the inter-vehicle relative pose using a monocular camera. Let $F_{E}$ denote the leader body frame and $F_{C}$ the follower camera frame. The relative pose to be estimated is

(1) $$T_{E}^{C}=\left[\begin{array}{cc} R & t\\ 0^{⊤} & 1 \end{array}\right]\in \mathrm{SE}\left(3\right),$$

which maps a point PE in the leader frame into the follower camera frame according to PC=RPE+t.

The vector-light marker is generated by four collimated blue lasers installed inside the leader AUV. Their beams pass through hull-flush outlets and are constrained to a common transverse plane perpendicular to the vehicle’s longitudinal axis. Two pairs of beams with different directions intersect in this plane, forming four feature points: $P_{1}$ (top), $P_{2}$ (right), $P_{3}$ (left), and $P_{4}$ (bottom), as shown in Figure 1b,c. The camera of the trailing follower faces this transverse plane. The four beams, made visible by scattering, and their intersections therefore form a stable structured marker in the image. The coordinates of the intersections in $F_{E}$ are obtained by offline calibration of the outlet positions, beam directions, and intersection geometry. No physical target surface is added outside the hull. Instead, the visible beam paths in water constitute the observable geometry: beam directions provide linear structures, while their spatial intersections provide point constraints. Together, they establish the 3D–2D correspondences required for monocular pose estimation and provide an explicit geometric basis for subsequent multi-candidate pose generation.

Assembly and calibration must ensure that the intersections are physically repeatable. The intersection of two projected beams in the image corresponds to a fixed 3D point only if the associated spatial beams actually intersect. The two beam pairs are therefore aligned to intersect during assembly, and the minimum spatial distance between each corresponding pair is verified during calibration. In the prototype, the four intersections form an approximately rhombic pattern in the z=0 plane of $F_{E}$, with a horizontal span of $P_{2}P_{3}=260$ mm and a vertical span of $P_{1}P_{4}=130$ mm. These dimensions are determined jointly by the available installation space in the 124 mm-diameter hull and the beam-intersection distance. On other platforms, the outlet directions and crossing geometry can be adjusted to the hull dimensions without changing the subsequent observation and pose-estimation pipeline. This fore–aft guidance configuration matches the line-of-sight geometry of following, formation keeping, and close-approach tasks and is used in all experiments.

### 3.2. Underwater Visibility and Beam-Image Degradation

The vector-light pattern is observable because of lateral scattering of laser light in water. The on-axis irradiance of a collimated laser after propagation over distance *z* approximately follows the Beer–Lambert law, $I\left(z\right)=I_{0}e^{-cz}$, where the attenuation coefficient c=a+b comprises the absorption coefficient *a* and scattering coefficient *b*. A wavelength of 450 nm is used to exploit the relatively low attenuation of water in the blue–green spectral band [53,54,55]. Scattering has a dual effect. It makes the beam visible to an off-axis camera but also introduces veiling light and reduces the contrast between the beam and its local background. Stable detection depends on the available contrast margin relative to image noise, exposure, and segmentation requirements.

The algorithm must therefore process degraded underwater beam observations rather than ideal geometric lines. Scattering halos can merge adjacent bright bands near their intersections, while thresholding may break weak distant responses. Bubbles and suspended particles produce isolated bright spots, and vehicle motion and exposure variations can broaden or weaken local responses. During threshold segmentation, connected-component analysis, and line detection, these factors readily cause false breaks, incorrect connections, and spurious segments. Local pixel-level connectivity is consequently unreliable. The proposed extraction method instead treats line segments as the basic units and aggregates them into complete beams according to their direction, transverse position, and axial gap.

### 3.3. Hough–Strip Structured Feature Extraction

**Figure 2:**
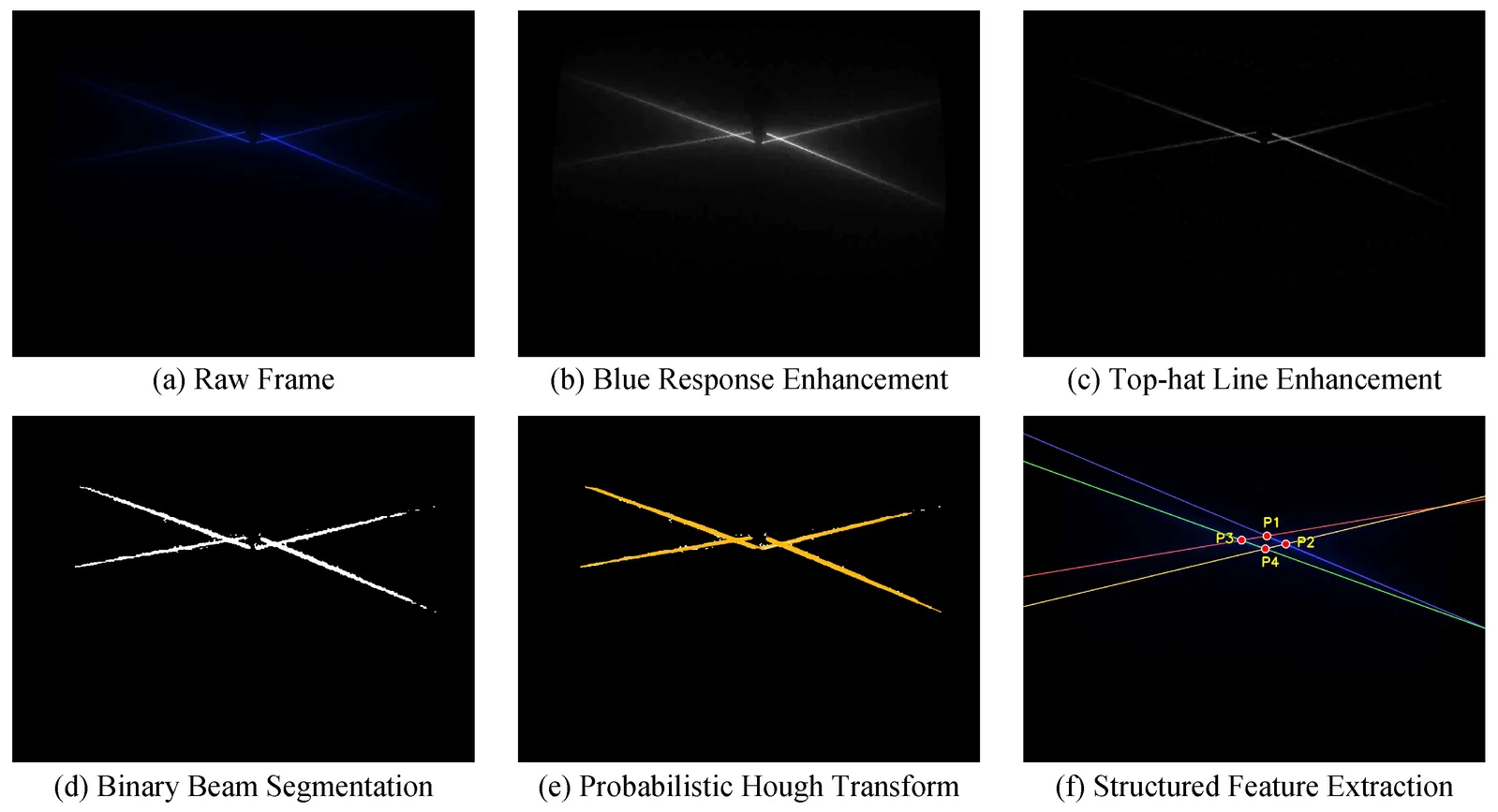
Intermediate results of structured vector-light feature extraction. All panels show the same real underwater frame: (**a**) undistorted raw image; (**b**) local-contrast and blue-response enhancement; (**c**) Top-hat line enhancement; (**d**) binary beam candidates obtained by thresholding and morphological processing; (**e**) line segments detected by the probabilistic Hough transform; and (**f**) four principal beams and image intersections $p_{1}$–$p_{4}$ obtained by strip clustering and least-squares fitting. Labels $P_{1}$–$P_{4}$ indicate the corresponding 3D feature identities.

The follower must recover the four principal beams and their intersections from real underwater images (Figure 1d). Feature recovery is organized as a structured sequence: enhance the principal ridges, detect candidate segments, merge segments from the same beam, and compute the intersections. Figure 2a–f presents the intermediate results for a real underwater frame, and Algorithm 1 summarizes the complete procedure. A valid-region mask $M_{v}$ is generated in advance from the fixed field of view after camera undistortion. It excludes black undistortion borders and regions occluded by the follower’s own hull. Contrast-limited adaptive histogram equalization (CLAHE) is first applied within $M_{v}$ to reduce the effect of nonuniform ambient illumination [56]. Because the laser energy is concentrated in the blue band, an opponent response is then constructed as

(2) $$I_{b}=\max\;\left(0,\;B-\alpha (R+G)\right),$$

where α is the color-suppression weight. After Gaussian filtering suppresses shot noise, the blue beams stand out clearly from the background (Figure 2b). A morphological Top-hat transform then extracts narrow responses brighter than the local background [57]. This operation suppresses slowly varying scattering halos and balances strong near-field beams against weak distant beams (Figure 2c). Percentile thresholding followed by morphological closing and opening produces a binary map of candidate beams (Figure 2d). Closing bridges short gaps, whereas opening removes isolated noise. The percentile threshold is applied to the Top-hat response rather than the raw grayscale image, so beam responses are concentrated in the extreme upper tail of the distribution. Even when near-field beams occupy many pixels, closing can bridge small local gaps introduced by threshold truncation.

**Algorithm 1** Structured vector-light feature extraction (single frame)**Input:** 
Undistorted RGB image I=(R,G,B); valid-region mask $M_{v}$**Output:** 
Image features $\left\{p_{1},p_{2},p_{3},p_{4}\right\}$ or an extraction-failure flag  1:Apply CLAHE local-contrast enhancement within $M_{v}$  2:$I_{b}\leftarrow \max\left(0,B-\alpha \left(R+G\right)\right)$, followed by Gaussian filtering  3:$I_{t}\leftarrow \mathrm{TopHat}\left(I_{b}\right)$ to suppress slowly varying halos and enhance line responses  4:Apply percentile thresholding within $M_{v}$; use closing to bridge gaps and opening to remove noise  5:Detect the line-segment set $S=\{s_{j}\}$ using the probabilistic Hough transform  6:Cluster segments from the same beam under the strip constraints in Equation (3)  7:Fit a principal line to each strip cluster by least squares and retain the four beams with the greatest cumulative lengths  8:Order the principal lines by slope sign and position, then intersect them to obtain $p_{1}$–$p_{4}$  9:**if** Too few lines are found or an intersection lies outside the valid image region **then**10:    **return** extraction-failure flag11:**end if**12:**return** $\left\{p_{1},p_{2},p_{3},p_{4}\right\}$

The probabilistic Hough transform [58] detects a set of short line segments $S=\{s_{j}\}$ in the binary candidate map (Figure 2e). Each segment is represented by its center $c_{j}$, unit direction $d_{j}$, endpoints, and length $L_{j}$. Bubbles, scattering highlights, and local occlusions often split a real underwater beam into multiple segments. Merging solely by the distance between infinitely extended lines can incorrectly combine adjacent beams, whereas connected-component merging may fail across gaps. Strip-constrained clustering is therefore used. Two candidate segments are assigned to the same physical beam only if their directional difference, transverse separation, and axial gap jointly satisfy

(3) $$\Delta \theta_{180}<\tau_{\theta },\;\left|{\bar{r}}_{i}-{\bar{r}}_{j}\right|<\tau_{\rho },\;g_{ij}<\tau_{g}.$$

Here, $\Delta \theta_{180}$ is the directional difference with 180° periodicity. Before segments *i* and *j* are compared, their direction signs are adjusted such that $d_{i}^{⊤}d_{j}\geq 0$. Their common direction and normal are then defined as $d_{ij}=\left(d_{i}+d_{j}\right)/∥d_{i}+d_{j}∥$ and $n_{ij}={\left(-d_{ij,y},d_{ij,x}\right)}^{⊤}$, respectively. The quantity $\bar{r}$ is the mean projection of a segment’s endpoints onto $n_{ij}$, and $g_{ij}$ is the shortest axial gap between the two intervals obtained by projecting the segments onto $d_{ij}$. After Top-hat enhancement and percentile segmentation, the high-response ridge near each beam center is confined to a narrow region. Accordingly, $\tau_{\rho }=5$ px is used at a resolution of 1440×1080. Equation (3) defines a strip with finite width and gap tolerance. It merges locally fragmented segments from the same beam while suppressing erroneous aggregation across adjacent beams. Least-squares fitting of the sampled points in each strip cluster yields the principal beam centerline. The four most prominent lines are selected by cumulative length and ordered by their slope signs and positions (Figure 2f), giving

(4) $$p_{1}=\ell_{1}\cap \ell_{3},\;p_{2}=\ell_{1}\cap \ell_{4},\;p_{3}=\ell_{2}\cap \ell_{3},\;p_{4}=\ell_{2}\cap \ell_{4}.$$

With an image line ℓ:ax+by+c=0 represented by the homogeneous line coordinates $\tilde{\ell }={\left(a,b,c\right)}^{⊤}$, an intersection is computed as the homogeneous cross product $\tilde{p}={\tilde{\ell }}_{a}\times {\tilde{\ell }}_{b}$. Extraction fails if fewer than four principal lines are available or any intersection falls outside the valid image region. The area, side-length ratio, and diagonal ratio of the rhombus formed by $p_{1}$–$p_{4}$ are then monitored. Their medians and median absolute deviations (MADs) are calculated over a 30-frame sliding window, and a frame is rejected if any statistic deviates from its median by more than three MADs. This geometric gate is the final output condition of structured observation recovery and is applied only once in the shared front end. Only image points that pass the gate are forwarded to pose estimation.

### 3.4. Camera Model and Underwater Calibration

The follower camera observes the marker through a flat port. Because of refraction at the flat interface, such a camera does not strictly follow the single-viewpoint pinhole model [59]. The intrinsic parameters were therefore calibrated offline in water from images of a calibration board captured at different distances and orientations [60], yielding effective intrinsic and distortion parameters that approximate the refractive effects under the calibration conditions [61]. The mean reprojection error of the underwater calibration, computed as the average Euclidean distance between the detected chessboard corners and their reprojections, was 0.43 px. No explicit refraction correction is applied; this in-water pinhole approximation is a modeling assumption of the present work.

## 4. Temporally Continuous Planar Relative Pose Estimation

This section describes how the 6-DoF relative pose is recovered from the 3D–2D correspondences produced by structured extraction, corresponding to the pose-estimation and closed-loop feedback stage in Figure 1e. Let K denote the camera intrinsic matrix. The four coplanar feature points have coordinates PiE=(Xi,Yi,0) in the z=0 plane of $F_{E}$, and their image observations are denoted by ${\hat{p}}_{i}$. They satisfy the pinhole model sip^˜i=K(RPiE+t). The *x*, *y*, and *z* axes of the camera frame point rightward in the image, downward in the image, and forward from the camera, respectively. To align the attitude representation with the physical rotation axes in this frame, a Z–Y–X sequence is used: $R=R_{z}\left(\phi \right)R_{y}\left(\psi \right)R_{x}\left(\theta \right)$. Here, ϕ, ψ, and θ denote roll about the optical *z*-axis, yaw about the vertical *y*-axis, and pitch about the horizontal *x*-axis, respectively. All attitude errors, dynamic differences, and tracking curves follow this convention.

Because only four coplanar features are observed, image blur and intersection-localization errors are amplified in depth and attitude estimates. In addition, the coplanar configuration may generate two pose branches with similar reprojection errors (Section 2). Selection based solely on the current reprojection error can switch between these branches. Each selected pose may fit its own frame well, yet the resulting sequence can exhibit pronounced attitude jumps. A pose initialization consistent with the current observation is therefore first constructed from the planar homography and refined by LM. IPPE candidates and the previously accepted pose provide additional initializations. Candidate selection first enforces consistency with the current image and then uses inter-frame motion continuity to determine the output, so that the temporal prior does not override the current visual evidence. Algorithm 2 summarizes the procedure.
**Algorithm 2** Temporally continuous vector-light relative pose recovery**Input:** 
Image points $p_{1}$–$p_{4}$ passing the front-end geometric gate; 3D points PiE; previously accepted poses**Output:** 
Current-frame pose $\left(R_{k},t_{k}\right)$ or a rejection flag  1:Obtain an initialization by planar-homography decomposition and refine it by LM reprojection minimization  2:Refine the previous pose and the IPPE solutions separately as additional LM initializations  3:Construct an observation-consistent candidate set using the minimum reprojection error  4:Predict $\left(R_{k|k-1},t_{k|k-1}\right)$ from the preceding pose or a constant-velocity model  5:Evaluate Equation (7) for the observation-consistent candidates and select the minimum-cost candidate  6:Accept and store $\left(R_{k},t_{k}\right)$, and update the pose history  7:**return** $\left(R_{k},t_{k}\right)$

### 4.1. Planar Initialization and Reprojection Refinement

Let ${\bar{p}}_{i}=K^{-1}{\tilde{\hat{p}}}_{i}$ be a normalized image point. The homography $H=[\;h_{1}\;h_{2}\;h_{3}\;]$ relates a planar point $\left(X_{i},Y_{i}\right)$ to this observation as ${\bar{p}}_{i}≃H{\left[X_{i},Y_{i},1\right]}^{⊤}$. After H is solved from the four correspondences, the rotation columns and translation are obtained as

(5) $$\lambda =\pm \frac{2}{∥h_{1}∥+∥h_{2}∥},\;r_{1}=\lambda h_{1},\;r_{2}=\lambda h_{2},\;t=\lambda h_{3},\;r_{3}=r_{1}\;\times \;r_{2}.$$

The sign of λ is chosen such that $t_{z}>0$, which is equivalent to selecting the appropriate global sign of H because a homography is defined only up to a nonzero scale. The matrix $R_{0}=\left[r_{1}\;r_{2}\;r_{3}\right]$ is then orthogonally projected onto SO(3) by singular value decomposition, with its determinant constrained to +1 [60]. Starting from this initialization, LM optimization [62] minimizes the mean squared reprojection error of the four points,

(6) Erep(R,t)=14∑i=14πK(RPiE+t)−p^i22,

and produces a candidate pose for the current frame. To match the pixel units used in the experimental tables, the per-frame reprojection error is defined as $e_{\mathrm{rep}}=\sqrt{E_{\mathrm{rep}}}$. This closed-form planar-homography initialization followed by LM reprojection refinement constitutes the basic stage of the proposed solver. In the ablation of Section 5.4, this stage alone is referred to as the single-initialization variant, whereas the complete solver used in all accuracy and closed-loop experiments is referred to as “Proposed.” Like conventional iterative PnP, this stage minimizes the reprojection error. It differs in using planar-homography construction and rotation orthogonalization as an explicit, controllable initialization and then extending this design to multi-start candidate generation.

Within the normal observation range, small changes in the intersections between adjacent frames generally produce only small changes in the homography initialization. LM refinement therefore remains on the same convergence branch in most frames; however, as the single-initialization results in Section 5.4 show, branch switching still occurs in near-frontal frames. To further reduce switching when the two branches have similar reprojection errors, the previously accepted pose and the planar candidates returned by IPPE are also refined by LM. Together with the homography-initialized solution, they form the pose-candidate set $C_{k}=\left\{\left(R_{k}^{\left(j\right)},t_{k}^{\left(j\right)}\right)\right\}$. This multi-start strategy broadens candidate coverage without altering either the observation model or the refinement objective.

### 4.2. Temporally Continuous Candidate Selection

To suppress inter-frame jumps caused by the planar two-solution ambiguity, the candidate set is first constrained by the current observation. Let $e_{\min}=\min_{j}e_{\mathrm{rep}}^{\left(j\right)}$. Only observation-consistent candidates satisfying $e_{\mathrm{rep}}^{\left(j\right)}\leq e_{\min}+\delta_{e}$ are retained, with $\delta_{e}=1$ px. If accepted poses are available for the two preceding frames, a constant-velocity model predicts the current reference pose. Translation is predicted as $t_{k|k-1}=2t_{k-1}-t_{k-2}$, and rotation is predicted by composing the previous rotation with the most recent relative rotation increment. With only one previous pose, that pose is used directly as the reference; the candidate in the first frame is selected by reprojection error alone. The cost for each observation-consistent candidate is

(7) $$J_{k}^{\left(j\right)}=w_{e}e_{\mathrm{rep}}^{\left(j\right)}+w_{R}d_{R}\;\left(R_{k}^{\left(j\right)},R_{k|k-1}\right)+w_{t}{∥t_{k}^{\left(j\right)}-t_{k|k-1}∥}_{2},$$

where $e_{\mathrm{rep}}^{\left(j\right)}$ is the per-frame root-mean-square reprojection error in pixels, $d_{R}\left(R_{1},R_{2}\right)=\cos^{-1}\;\left(\left(\mathrm{tr}\left(R_{1}^{⊤}R_{2}\right)-1\right)/2\right)$ is the rotational distance in radians, and translation differences are measured in centimeters. The weights are $w_{e}=1.0\;{\mathrm{px}}^{-1}$, $w_{R}=0.5\;{\mathrm{rad}}^{-1}$, and $w_{t}=0.1\;{\mathrm{cm}}^{-1}$; that is, 1 px of reprojection error costs as much as 2 rad of rotational deviation or 10 cm of translational deviation. Because the cost is evaluated only among candidates whose reprojection errors lie within $\delta_{e}$ of the best fit, the motion terms decide among solutions that explain the current image almost equally well, as occurs in near-frontal frames, and do not override clear image evidence. The bandwidth $\delta_{e}=1$ px retains the candidate supported by the current observation while excluding candidates with clearly larger reprojection errors. These values were set according to the units and scales of the respective terms, fixed before the evaluation, and kept unchanged in all experiments; their sensitivity is examined in Section 5.4. Equation (7) introduces motion continuity only among candidates supported by the current observation. The candidate with the smallest $J_{k}^{\left(j\right)}$ is selected as the output. This procedure is termed temporally continuous candidate selection (TCS).

Previously accepted poses provide additional initializations for LM refinement (Section 4) and the motion reference in Equation (7). Each output is one of the candidates refined against the current image observations, with a reprojection error within $\delta_{e}$ of that of the best-fitting candidate; output poses are not averaged across frames. The candidates differ mainly in attitude, often by tens of degrees (Section 5.4), so the selection mainly determines which of these distinct solutions is output.

Recovery from an incorrect branch is conditional. After a candidate has been selected in an ambiguous frame, the output switches to another candidate only if the selected branch leaves the observation-consistency band, i.e., its reprojection error exceeds $e_{\min}+\delta_{e}$, or if its total cost in Equation (7) exceeds that of another observation-consistent candidate. Such changes can occur as the viewpoint changes; the output then follows the candidate best supported by the current observation and the motion reference, which does not guarantee that the physically correct branch is recovered. When the front end rejects two consecutive frames, the prediction history is cleared, and the next valid frame is initialized according to reprojection error. If both branches retain nearly identical reprojection errors over an extended interval, the monocular coplanar observation itself cannot determine the physically correct solution. In this case, the system can only maintain a temporally consistent output, which is an inherent limit on the applicability of this class of methods. Pose recovery uses only the current-frame observation and previously accepted poses; no retrospective trajectory smoothing is performed. The front-end geometric gate is applied identically to all solvers and is excluded from the subsequent ablation comparison. Throughout this paper, “geometric gate” denotes this front-end check of the rhombus geometry, and “observation-consistency band” denotes the $\delta_{e}$ criterion in TCS.

## 5. Experiments and Results

### 5.1. Experimental Platforms and Evaluation Protocol

**Figure 3:**
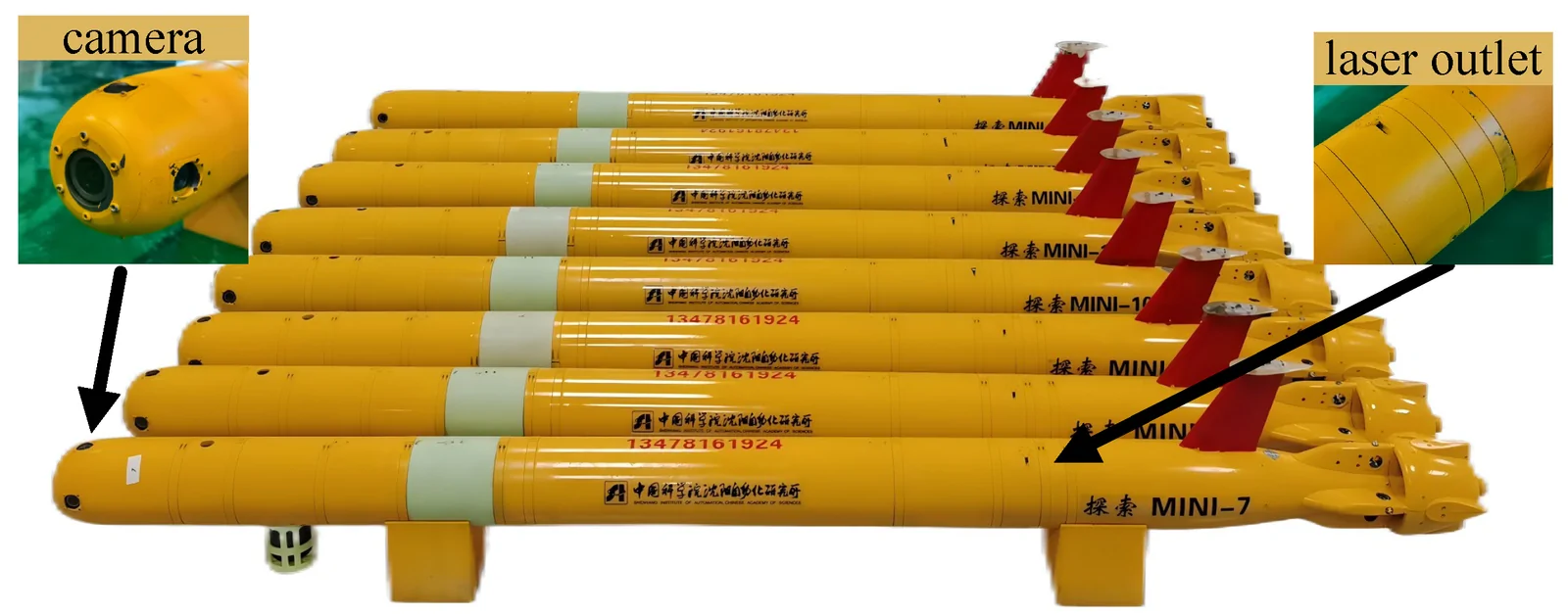
Hardware implementation of the TS MINI autonomous underwater vehicles (AUVs) and active vector-light marker. Four blue lasers are installed inside the leader AUV. Their beams pass through hull-flush outlets and form a visible crossed marker in a common transverse plane perpendicular to the longitudinal axis. A monocular camera is mounted in the bow of the follower AUV, and image processing and pose estimation run on an onboard Jetson TX2.

The experimental system consists of two identical TS MINI AUVs developed by the Shenyang Institute of Automation, Chinese Academy of Sciences, each with a hull diameter of 124 mm (Figure 3). Four 450 nm collimated blue lasers are installed inside the leader, each with a rated optical power of 1 W. The beams pass through hull-flush outlets and form a crossed marker in a common transverse plane perpendicular to the longitudinal axis. No external emitter arm, forward target board, or protruding guidance structure is used. A 1440×1080 monocular camera is mounted in the bow of the follower. Image acquisition, vector-light extraction, and pose estimation all run on an onboard Jetson TX2. For each trial, the beam attenuation coefficient of the water was measured on site using a WET Labs C-Star transmissometer with a 25 cm path length at 470 nm [63]. The fixed-relative-pose accuracy experiments (hereafter, fixed-pose tests) were conducted in an indoor test pool, where $c=0.5\;m^{-1}$. Structured extraction evaluation, dynamic-sequence comparison, and closed-loop tracking were conducted at Qiandao Lake in Hangzhou, Zhejiang Province, China, where $c=1.1\;m^{-1}$.

Table 1 lists the principal processing parameters used consistently throughout the experiments. The percentile segmentation threshold is determined adaptively from the response distribution within $M_{v}$ in the current frame. Geometric-gating thresholds are updated adaptively from robust statistics over a sliding window, whereas strip clustering and Hough detection use fixed pixel and angular thresholds. These thresholds are set according to the beam geometry at the working resolution: $\tau_{\rho }$ matches the width of the enhanced beam ridge, $\tau_{\theta }$ is smaller than the angular separation between the two beam families, $\tau_{g}$ covers the gaps along fragmented beams, and the percentile threshold reflects the small fraction of beam pixels in the valid region.

At a resolution of 1440×1080 on the Jetson TX2, image enhancement and morphological processing require approximately 47 ms on average. Hough–strip extraction and intersection recovery require approximately 41 ms, and candidate pose estimation with TCS requires approximately 6 ms. The total per-frame processing time, including data transfer, is approximately 96 ms. The 5 Hz output rate used in the experiments is set by the common camera-acquisition and control period and does not represent the computational limit of the platform.

**Figure 4:**
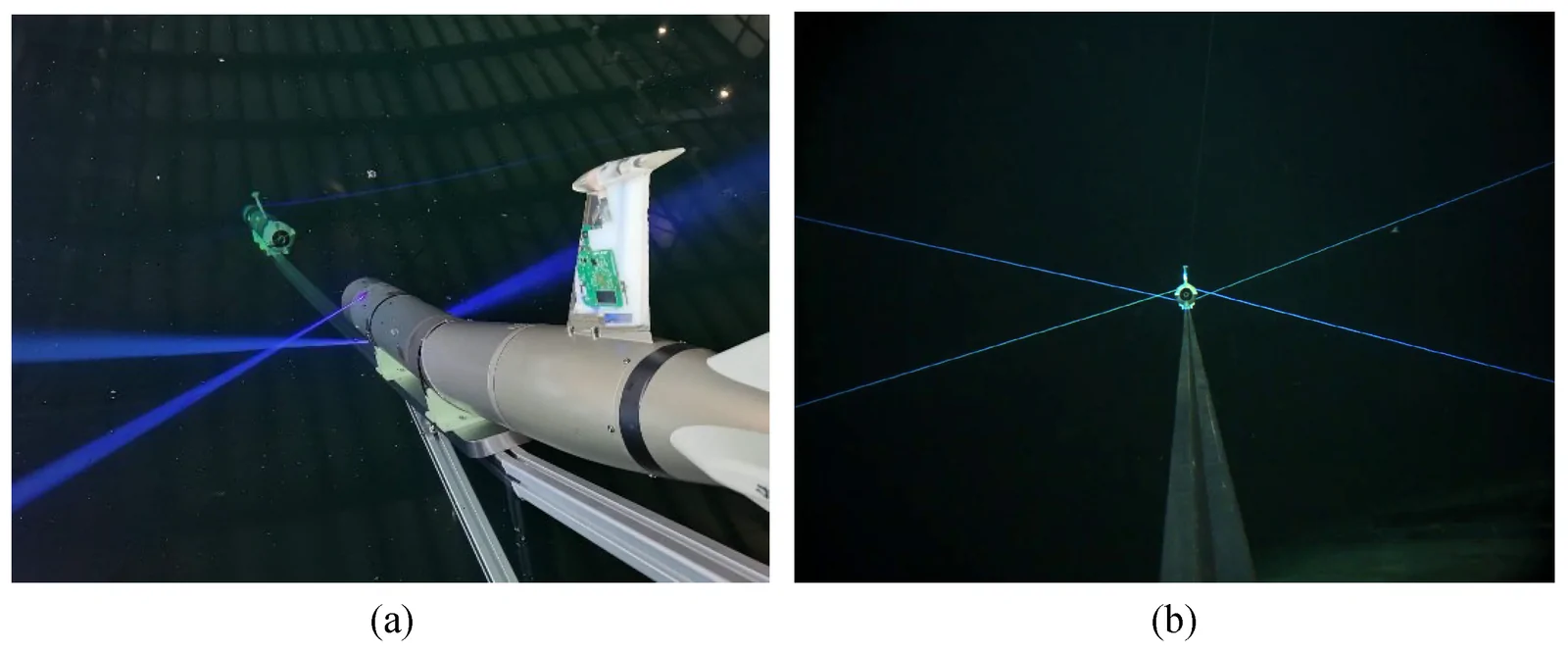
Setup for the fixed-pose tests. (**a**) External view: the leader and follower AUVs are fixed to a common rigid frame, with the follower facing the leader’s vector-light marker. Their reference relative pose remains constant while the entire frame can translate and oscillate in the water. (**b**) Onboard view from the follower camera showing the two pairs of intersecting beams emitted by the leader.

The experiments evaluate four levels of performance: observation recovery, static accuracy, temporal consistency, and closed-loop tracking, corresponding to Section 5.2, Section 5.3, Section 5.4 and Section 5.5. The fixed-pose tests quantify the accuracy of the sensing system against a mechanical reference, whereas the effect of the proposed solver, which targets pose jumps under dynamic imaging conditions, is examined on dynamic lake data. In the fixed-pose tests, the leader and follower were attached to a common rigid frame in the test pool (Figure 4). A total of 3000 frames were acquired, with 1000 frames at each distance of 2, 4, and 6 m. All frames were processed with the complete proposed method, i.e., the same configuration as in the closed-loop experiment. The reference relative pose was determined before the experiment from the translation and rotation between the follower’s camera frame and the leader’s body frame, measured with respect to the mechanical installation datum. During acquisition, the entire rigid frame translated and oscillated in the water to introduce realistic background, water, and imaging disturbances while preserving the inter-vehicle relative pose.

### 5.2. Structured Feature Extraction Performance

To evaluate the Hough–strip extraction pipeline on degraded real underwater images, several contiguous subsequences were taken from multiple dynamic videos recorded during lake trials. In their original temporal order, they form a front-end evaluation set of 2000 frames covering different backgrounds and imaging disturbances. The 30-frame sliding statistics used for geometric gating were maintained independently within each subsequence and reinitialized when the subsequence changed, preventing statistics from different distances or viewpoints from being mixed. All samples were processed with the same parameters listed in Table 1. Frame validity was evaluated at two levels. Detection was considered successful when all four principal beams and intersections $p_{1}$–$p_{4}$ were recovered. All detections in the evaluation set were verified manually frame by frame; a detection was counted as successful only if the four fitted lines coincided with the four beams visible in the image. A frame then passed the geometric gate only if the rhombus area, side-length ratio, and diagonal ratio all satisfied the sliding-window median–MAD criterion.

Table 2 reports a detection success rate of 99.85% (1997 frames) for the proposed Hough–strip method. Of these frames, 1988 also passed the geometric gate, so valid front-end observations account for 99.40% of all frames; 3 frames failed detection and 9 were rejected by the gate. The failed and rejected frames mainly contain excessively weak beam responses, principal-line errors caused by local highlights, or abnormal geometric ratios among the intersections. The Hough–strip pipeline thus provides structurally complete four-beam observations across different dynamic underwater subsequences, while the geometric gate intercepts a small number of anomalous inputs before pose estimation.

**Figure 5:**
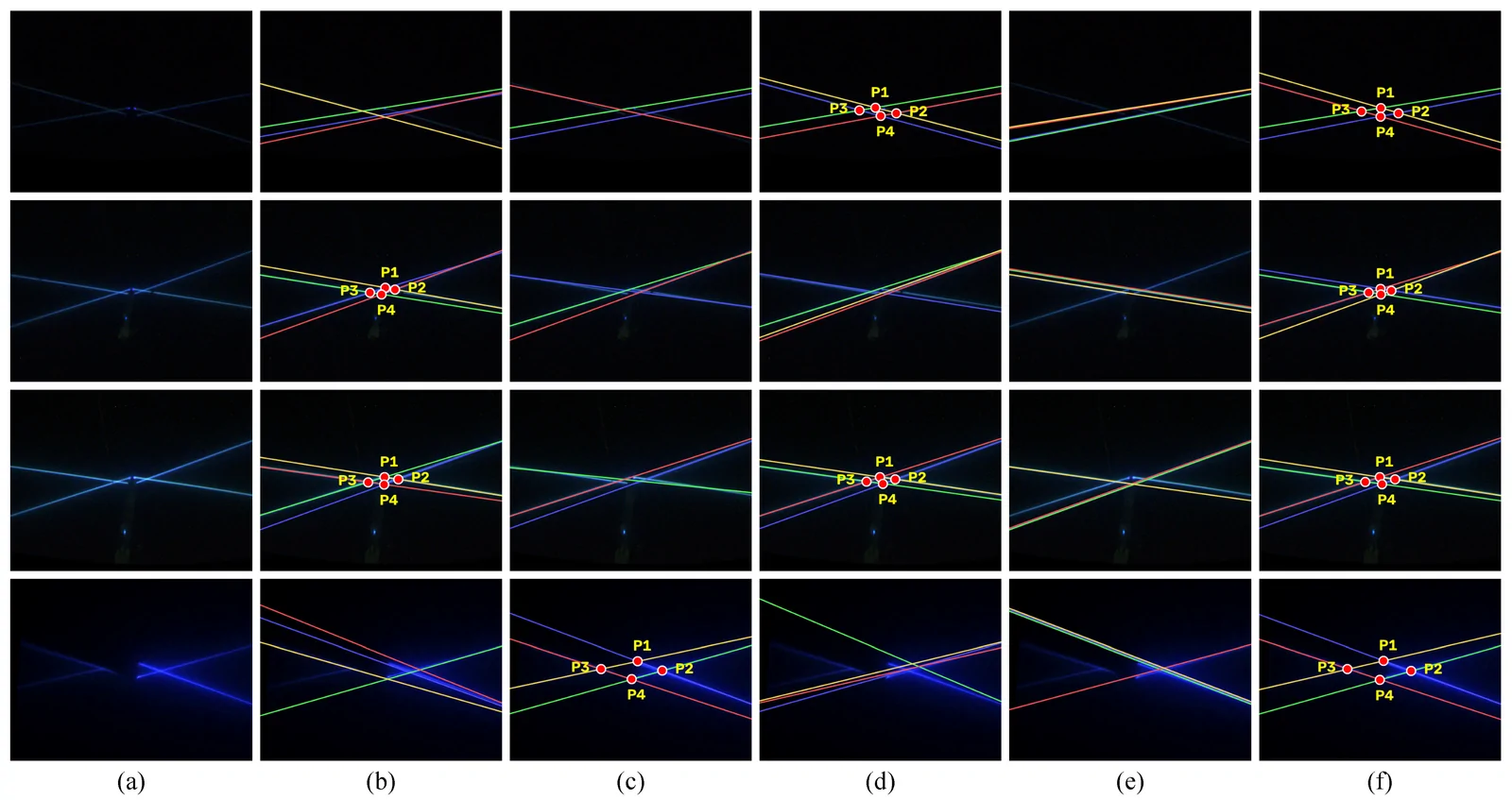
Principal-beam and intersection recovery by different line-extraction methods on representative degraded underwater frames. (**a**) Undistorted raw image; (**b**) standard Hough transform; (**c**) sequential RANSAC line fitting; (**d**) LSD segment detection; (**e**) Hough segments merged without strip clustering; (**f**) proposed Hough–strip method. Colored lines are the recovered principal beams, and red dots mark the image intersections, labeled by their feature identities $P_{1}$–$P_{4}$; missing dots indicate that the method did not obtain all four intersections. Each row shows the same local region around the intersections.

To evaluate the contribution of the Hough–strip design, Figure 5 and Table 2 compare five line-extraction methods applied to the same enhanced binary beam maps: (b) the standard Hough transform, which selects the four highest-voted lines in the (ρ,θ) space [58]; (c) sequential RANSAC line fitting [50], which repeatedly fits the line with the most inliers and removes its pixels before searching for the next line; (d) the line segment detector (LSD) [64], whose segments are merged into four lines according to collinearity; (e) the Hough segments of the proposed pipeline merged by the distance between their extended lines instead of by strip clustering; and (f) the proposed Hough–strip method. Stages shared with the proposed pipeline use the settings listed in Table 1: all methods take the same enhanced binary beam map, and method (e) uses the same probabilistic Hough segments. The remaining method-specific parameters, such as the inlier and merging tolerances, were set according to the beam width and the angular separation between the two beam families at the working resolution, on the same geometric basis as the thresholds in Table 1.

Figure 5 shows four representative degraded frames. The standard Hough transform, RANSAC, and LSD recover the four intersections in some of these frames but not in others, and merging without strip clustering does not recover them in any of the four frames. In most failed cases, several of the recovered lines coincide with the same beam or with beams of the same family, so that four valid intersections cannot be formed. The Hough–strip method recovers all four intersections in the four frames. Over the full evaluation set, its success rate is 99.85%, whereas those of the baselines range from 33.25% to 91.35% (Table 2). Since all methods share the same input, the difference arises from how line evidence is grouped into beams: the strip constraint in Equation (3) groups segments jointly by direction, transverse position, and axial gap, which allows fragments of one beam to be merged while keeping nearly parallel beams of the same family apart.

### 5.3. Relative Pose Accuracy

**Figure 6:**
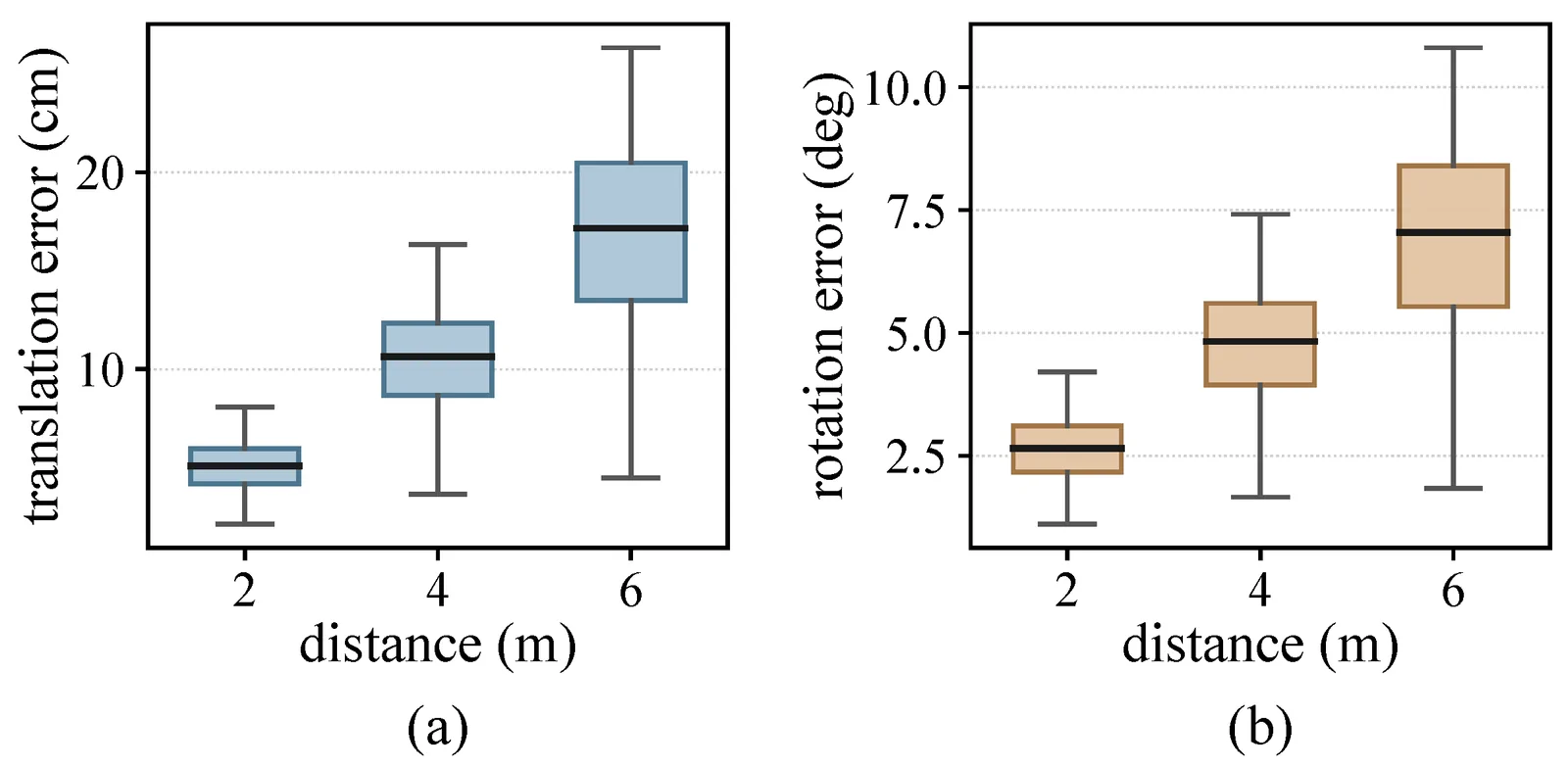
Box plots of the total per-frame errors of the proposed method in the fixed-pose tests. (**a**) Total translation error. (**b**) Total rotation error. The three boxes in each group correspond to observation distances of 2, 4, and 6 m. Each box spans the interquartile range, the horizontal line indicates the median, and the whiskers extend to 1.5 times the interquartile range.

Table 3 reports the root-mean-square error (RMSE) of each error component for the proposed method at 2, 4, and 6 m. Table 3 thus characterizes the accuracy of the sensing system, operated with the complete proposed method, against the mechanical reference under fixed-pose conditions. Figure 6 shows the corresponding box plots of the total per-frame errors. Both the componentwise errors and the dispersion of the boxes increase with observation distance. At longer distances, the marker subtends a smaller angle, amplifying the effect of intersection-localization errors on the estimated pose. The few outliers at each distance are of limited magnitude, indicating that the structured observations yield stable frame-level estimates.

For translation, the lateral and vertical (*x*, *y*) errors remain at the centimeter level at all three distances and are still within a few centimeters at 6 m. The optical-axis (*z*) error is substantially larger and dominates the total translation error (Figure 6a), because depth is constrained indirectly by the imaged scale of the marker and is more sensitive to image-point errors than lateral position. The depth RMSE increases from 4.8 cm at 2 m to 16.8 cm at 6 m, while the normalized depth error $e_{z}/Z$, with *Z* the reference range, rises from 2.4% to 2.8%. Over this interval, the depth error thus grows approximately linearly with range, slightly faster than a strictly linear trend, while the relative error remains between 2.4% and 2.8%. Calibration residuals in the beam directions and 3D intersections, principal-beam extraction bias, and random image-point errors may all contribute to this trend. The three tested distances are insufficient to separate these sources further. Nevertheless, in these tests the system kept the normalized optical-axis RMSE between 2.4% and 2.8% over 2–6 m without an external target surface or protruding light mount.

**Uncertainty of the reference.** The outlet seats of the laser emitter are machined by computer numerical control (CNC) with a specified positional accuracy and bore coaxiality both better than 0.01 mm. The resulting beam-pointing error is estimated to be less than 0.03°, corresponding to a positional error of the 3D intersections of less than 0.1 mm. The two vehicles are mounted on the rigid frame with a positional accuracy better than 1 mm, corresponding to an estimated angular error of less than 0.1°. These specifications indicate the nominal precision of the reference setup but do not constitute a propagated uncertainty budget; the errors in Table 3 are measured against this reference and therefore include contributions from both the estimation system and the reference setup.

For attitude, all three RMSE components are at the degree level and consistently satisfy roll<yaw<pitch (Table 3). This ordering is consistent with the difference in observability that arises because the horizontal span of the marker is larger than its vertical span. Roll is constrained by rotation of the entire rhombus in the image plane and has the largest lever arm. Yaw benefits from the greater horizontal span, whereas pitch is limited by the smaller vertical span and exhibits the greatest dispersion. Consequently, pitch dominates the total rotation error in Figure 6b.

### 5.4. Pose-Solver Comparison and Ablation on a Dynamic Sequence

**Figure 7:**
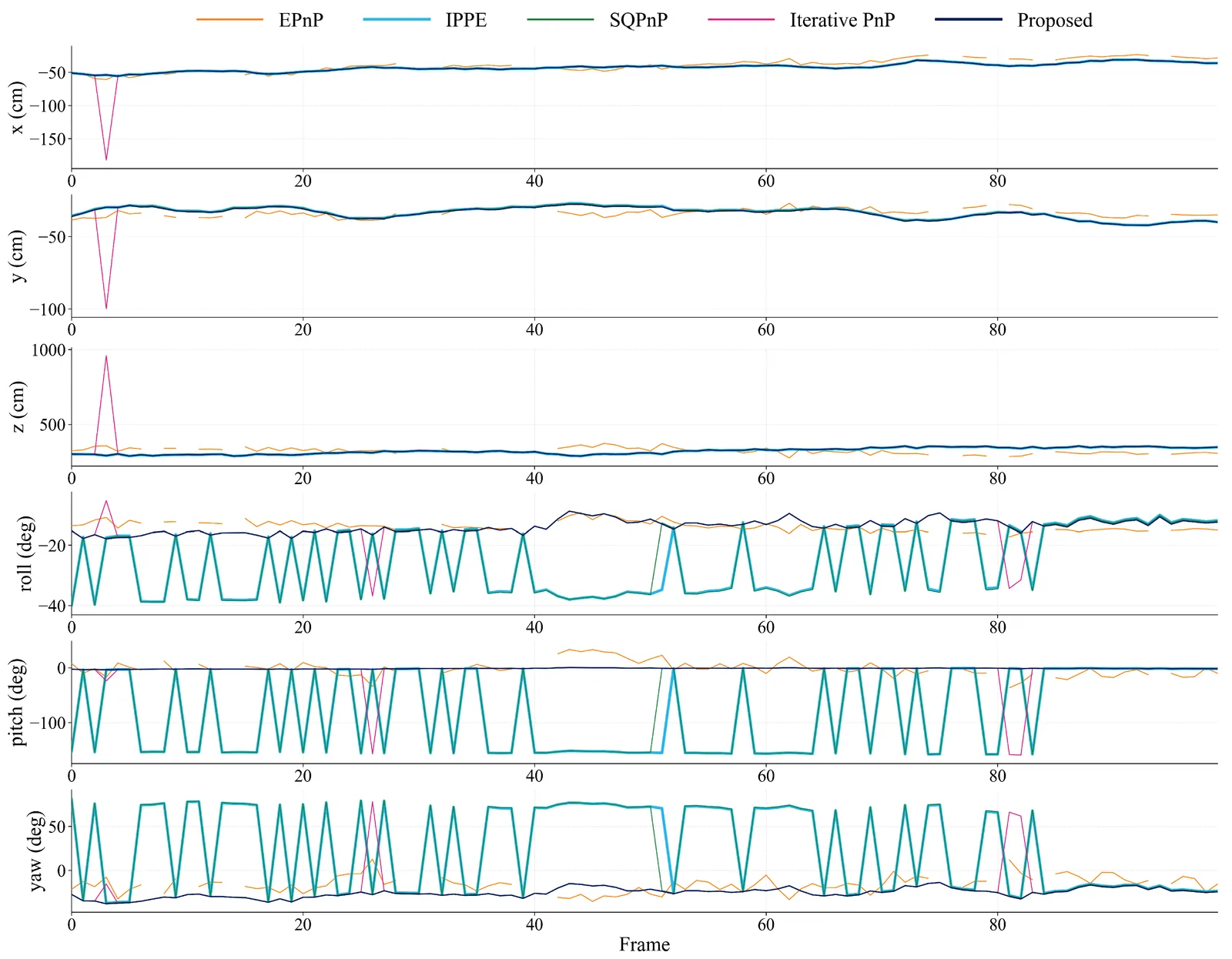
Pose components estimated by EPnP, IPPE, SQPnP, iterative PnP, and the proposed solver over 100 consecutive frames (approximately 20 s at 5 Hz) of dynamic lake data: from top to bottom, *x*, *y*, and *z* (cm) and roll, pitch, and yaw (°). All methods receive identical four-point observations. Gaps indicate invalid estimates or estimates far outside the plotted range. A line that is not visible is overlapped by the lines drawn over it; in particular, the green line inside the wide cyan band indicates overlapping IPPE and SQPnP estimates.

Independent 6-DoF ground truth was unavailable for the dynamic lake sequence used in this comparison. The solvers are therefore compared in terms of observation fit and temporal consistency; fixed-pose accuracy is reported separately in Section 5.3. Three metrics are used: the median and 95th-percentile reprojection errors, which measure the fit to the current four-point observation; the RMS of the third-order difference $\Delta^{3}q_{k}=q_{k}-3q_{k-1}+3q_{k-2}-q_{k-3}$, computed separately for the translation and Euler-angle vectors, which removes locally quadratic motion and emphasizes abrupt changes; and the translation and attitude jump rates, i.e., the percentages of transitions between adjacent valid frames in which any translation component changes by more than 5 cm or any Euler angle changes by more than 5°, respectively. These thresholds lie above the frame-to-frame fluctuation of a continuously tracked pose, whereas the jumps in Figure 7 involve Euler-angle changes exceeding 100° between adjacent frames. The jump rates therefore count abrupt changes and are not measures of accuracy; the zero-jump results refer to these thresholds. Euler angles are unwrapped with a 2π period, and all statistics are computed within contiguous valid segments with identical thresholds for every method.

To isolate pose solving, every method receives exactly the same four-point observations from a shared structured-extraction and geometric-gating front end. A solution is considered valid only if the solver returns finite R and t, all four reference points have positive depths in the camera frame, and $e_{\mathrm{rep}}<100$ px (approximately 5.6% of the image diagonal). The valid-solution rate uses the number of frames delivered by the shared front end as its denominator. The 100 px validity threshold was fixed before the methods were compared. It only identifies solver failures or clear outliers; it is neither an accuracy criterion nor part of candidate ranking. Failures in Table 4 therefore arise from the solver rather than from the shared front end. The comparison uses an independent, temporally continuous real-water sequence of approximately 2000 frames recorded during the lake trials, distinct from the multi-video front-end evaluation set in Section 5.2.

**Comparison with existing solvers.** Under the implementation and test conditions used here, EPnP showed substantially larger reprojection errors (median of approximately 90 px), a lower valid-solution rate (83.7%), and the highest jump rates. Given these large reprojection errors, its comparatively low attitude-jitter value should not be interpreted as evidence of superior estimation performance. IPPE, SQPnP, iterative PnP, and the single-initialization variant of the proposed solver all achieved median reprojection errors of approximately 3.5 px, indicating similar fits to the current image observations. They differed instead in the amplitude of high-frequency attitude fluctuations and in the frequency of jumps. After rounding, IPPE and SQPnP both yielded a third-order attitude difference of 311.4°, whereas their attitude jump rates were 13.09% and 6.62%, respectively. The third-order-difference RMS depends on the jump magnitude, whereas the jump rate counts only above-threshold events, so the two metrics need not rank the methods in the same order. Among these four methods, the single-initialization variant achieved the lowest third-order attitude difference (139.9°), suggesting that the explicit planar-homography initialization provides a suitable starting point for multi-start candidate generation.

**Pose jumps of representative solvers.** Figure 7 shows the six pose components estimated by the five methods from identical four-point observations over 100 consecutive frames (approximately 20 s at 5 Hz) of dynamic lake data. In most frames, the estimates of IPPE, SQPnP, iterative PnP, and the proposed solver closely match one another and form a common continuous trajectory. IPPE, SQPnP, and iterative PnP repeatedly deviate from this trajectory, mainly in attitude: the pitch, roll, and yaw estimates change by approximately 155°, 20–25°, and 100°, respectively, and some deviations persist for more than ten consecutive frames. The deviations of iterative PnP occur at different frame indices from those of IPPE and SQPnP. EPnP roughly follows the other methods but shows frequent gaps, corresponding to invalid estimates or estimates far outside the plotted range. The estimates of the proposed solver remain on the common trajectory throughout the segment, including the frames in which the other solvers deviate.

In frames without deviations, the estimates of the proposed solver closely match those of IPPE, SQPnP, and iterative PnP. The deviations of these three solvers include Euler-angle changes exceeding 100° between adjacent frames (0.2 s). Such abrupt changes were not observed in the output of the proposed solver over this segment. Over the full evaluation sequence, the proposed solver achieved reprojection errors comparable to those of IPPE, SQPnP, and iterative PnP (Table 4). Because the jump rates count above-threshold changes between adjacent frames, a deviation contributes mainly at its onset and at its return rather than once for every deviating frame. In closed-loop control, such abrupt changes would disturb the feedback.

**Ablation of the proposed solver.** The single-initialization variant still produced translation and attitude jump rates of 10.07% and 12.57%, respectively. In near-frontal frames, or when the two branches have similar reprojection errors, refinement from a single initialization can converge to the adjacent branch. Refining multiple initializations but selecting the candidate with the smallest reprojection error barely changed the jump rates, and the third-order attitude difference increased to 146.2°. When the two branches fit the image almost equally well, the minimum-error choice is determined by image noise and may alternate between branches in consecutive frames; the third-order difference weights such alternation more heavily than isolated jumps. Within the observation-consistency band ($\delta_{e}=$ 1 px), the proposed solver selects the candidate with the lowest combined reprojection and motion-consistency cost in Equation (7). It reduced the RMS of the third-order attitude differences from 139.9° to 12.6°, approximately 1/11 of the single-initialization value. No translation or attitude jumps were recorded on this sequence under the 5 cm/5° thresholds. The median reprojection error, the third-order translation difference, and the valid-solution rate remained essentially unchanged. Because the two branches have similar translations and differ mainly in attitude, the reduction in the third-order difference is concentrated in the attitude channel. The observation-consistency band also limits the influence of historical information: a previous branch that no longer agrees with the current image is removed from the candidate set by its reprojection error.

Together, the two ablation rows indicate that, on this sequence, neither refinement from a single initialization nor minimum-error selection among multiple candidates suppressed branch switching. The improvement was obtained when multiple initializations were combined with observation-constrained, motion-consistent selection, and the reprojection errors remained comparable. Table 4 compares the proposed solver with representative PnP solvers and reports ablations within the proposed solver; it does not include comparisons of different solvers under an identical selection strategy or with temporal filtering methods.

**Parameter sensitivity.** Scaling all weights in Equation (7) by a common factor does not change the selection, so only the ratios $w_{R}/w_{e}$ and $w_{t}/w_{e}$ matter, and $w_{e}$ is kept fixed. The bandwidth $\delta_{e}$ and the weights $w_{R}$ and $w_{t}$ were each halved and doubled relative to their nominal values, one parameter at a time. Two metrics were evaluated, corresponding to the two opposite failure modes: the attitude jump rate, which increases if the temporal constraint is too weak to suppress branch switching, and the 95th-percentile reprojection error, which increases if the temporal constraint is so strong that candidates fitting the current image less well are selected. As listed in Table 5, both metrics were unchanged at the displayed precision in all six settings. All settings share the same candidate-generation procedure, and $\delta_{e}$, $w_{R}$, and $w_{t}$ act only on the selection among the resulting discrete candidates; they do not enter the LM refinement. The estimated poses therefore remain unchanged as long as a parameter change does not alter the selected candidate. Because competing candidates differ in attitude by tens of degrees (Figure 7), the tested variations were too small to alter the selection. Within the tested range, the results are therefore insensitive to the parameter values on this sequence.

### 5.5. Lake-Based Closed-Loop Tracking Experiment

**Figure 8:**
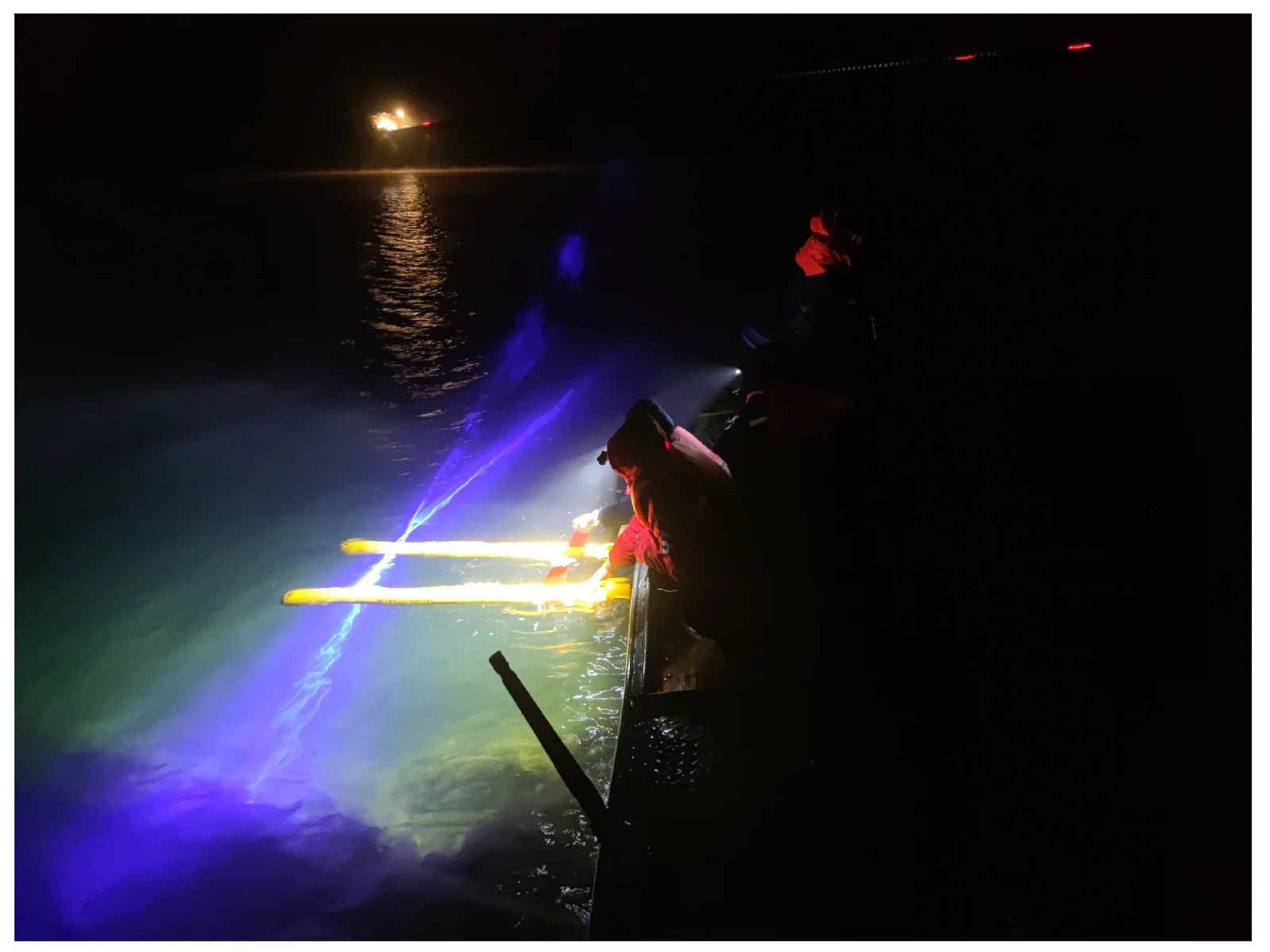
Deployment of the two TS MINI AUVs at the lake trial site. The four beams of the leader’s vector-light marker are visible propagating into the water column.

The closed-loop trial was conducted at night on an open lake. The two TS MINI AUVs were deployed from a support vessel as shown in Figure 8. Only the leader carries the vector-light marker; its four beams were switched on before entry and provided the guidance observation for the entire trial. The leader was released first and navigated ahead, after which the follower was released and tracked it using the onboard vector-light relative pose feedback alone.

The proposed method supplies the onboard controller with the relative state $\eta_{k}={\left[x_{k},z_{k},{\mathrm{yaw}}_{k}\right]}^{⊤}$, where *x* and *z* are expressed in centimeters and yaw in degrees. The desired state is $\eta_{d}={\left[0\;\mathrm{cm},350\;\mathrm{cm},0^{\circ }\right]}^{⊤}$. For the three channels, the error $e_{k}=\eta_{d}-\eta_{k}$ is regulated using the discrete proportional–integral–derivative (PID) law

(8) $$u_{i,k}=K_{P,i}\;e_{i,k}+K_{I,i}\sum_{j=0}^{k}e_{i,j}\Delta t+K_{D,i}\;\frac{e_{i,k}-e_{i,k-1}}{\Delta t},\;\Delta t=0.2\;s,$$

where i∈{x,z,yaw} indexes the channel, and $K_{P,i}$, $K_{I,i}$, and $K_{D,i}$ are its proportional, integral, and derivative gains. The gains were tuned empirically in preliminary in-water trials to balance tracking responsiveness, overshoot, and steady-state offset, and were then adjusted jointly with all three channels active to account for their coupling; they were kept fixed throughout the closed-loop experiment. The longitudinal range command is assigned to the main thruster. Lateral-centering and yaw commands are allocated through the stern vector-thrust mechanism, while the vehicle’s existing depth-hold loop maintains the vertical state. The *y*, pitch, and roll components are not used by this three-channel tracking controller, but remain available as part of the continuously reported 6-DoF relative state for attitude monitoring and future formation or docking tasks. The experiment therefore demonstrates three-channel closed-loop tracking rather than full 6-DoF closed-loop control.

To evaluate autonomous underwater tracking by the complete system, the vector-light relative pose was incorporated into the AUV control loop during the lake experiment. This implementation formed a closed-loop chain comprising vector-light visual observation, relative pose sensing, and motion control. Independent 6-DoF ground truth was not available in this trial. Closed-loop performance is therefore assessed from the target-centering process in the images, the convergence trends of the controlled states, and the continuity of the visual feedback. Deviations of the plotted trajectories from their reference lines are not interpreted as externally calibrated steady-state errors. Accordingly, this section evaluates closed-loop operation driven by the estimated pose and the continuity of the visual feedback, not independently verified 6-DoF tracking accuracy; quantitative accuracy statements are limited to the fixed-pose tests of Section 5.3.

**Figure 9:**
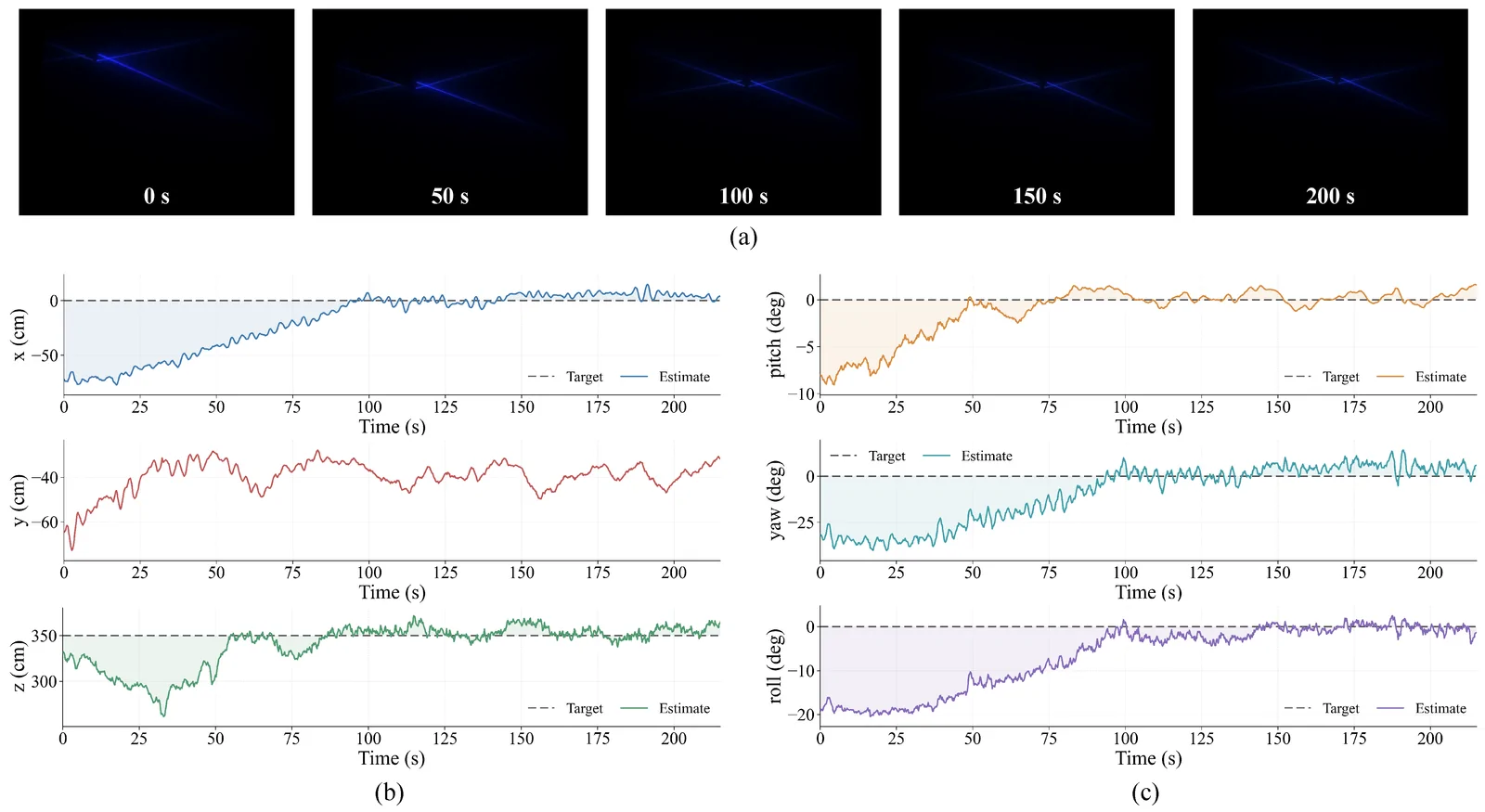
Observations and pose trajectories from the 214 s lake tracking experiment. (**a**) Raw underwater images acquired by the follower camera at 0, 50, 100, 150, and 200 s. The target moves gradually from the upper-left region toward the image center and remains there. (**b**) Time histories of the translation components x/y/z for 1065 valid poses. (**c**) Corresponding pitch/yaw/roll histories. Dashed lines for *x*, *z*, and yaw denote closed-loop references: z=350 cm and x=yaw=0. Zero lines for pitch and roll aid visualization of relative-attitude changes. The *y* component corresponds to the depth direction during depth-hold navigation and has no tracking reference.

The displayed segment contains 1071 input frames acquired at 5 Hz over approximately 214 s. The proposed method returns 1065 valid poses. The six invalid frames are neither plotted nor included in the trajectory statistics. Initially, the target lies in the upper-left region of the image; it then moves toward the center and remains there (Figure 9a). Correspondingly, the lateral offset *x* converges from approximately −70 cm to near zero. After an initial adjustment, the longitudinal range *z* stabilizes near its 350 cm reference, while yaw converges from approximately −30° to near zero (Figure 9b,c). The vertical position *y* remains bounded under depth-hold navigation. Relative pitch and roll gradually stabilize from initial values of approximately −9° and −19°, respectively. All six pose components remain continuous throughout the maneuver and agree with the target-centering process observed in the images.

Table 6 summarizes feedback continuity. The feature-detection success rate is 99.81%, the geometric-gate pass rate is 99.53%, and valid poses from the proposed method account for 99.44% of all frames. The longest observation gap is a single frame. In this nighttime trial at $c=1.1\;m^{-1}$, active vector-light observation, structured feature recovery, and temporally continuous pose estimation provided continuous feedback despite underwater imaging degradation, vehicle motion, and actuator disturbances, thereby supporting three-channel closed-loop tracking throughout the 214 s trial.

## 6. Conclusions

This paper presented a system for underwater relative pose estimation and autonomous tracking between AUVs based on a hull-integrated laser-beam marker. Four blue beams emitted through hull-flush outlets provide observations of four coplanar intersection points to a monocular camera without requiring an external optical target structure. Structured feature extraction and temporally continuous pose recovery convert degraded images into continuous feedback suitable for motion control. In fixed-pose pool tests over 2–6 m, the proposed method achieved centimeter-level lateral accuracy, normalized optical-axis RMSEs of 2.4–2.8%, and degree-level attitude accuracy. On dynamic lake data, the estimates of the proposed solver closely matched those of IPPE, SQPnP, and iterative PnP in frames without deviations, and the large attitude jumps observed for these solvers did not occur in its output. The proposed solver reduced the RMS of the third-order attitude differences by approximately one order of magnitude relative to the single-initialization variant at comparable reprojection errors, and no jumps were recorded under the 5 cm/5° thresholds. During lake-based closed-loop tracking, valid poses were obtained for 99.44% of the frames, and the estimated pose supported sustained three-channel closed-loop tracking of lateral position, following distance, and yaw during the 214 s nighttime trial.

This study covered the development of the sensing system: the marker was designed, fabricated, and integrated into a compact AUV hull; the extraction and pose-solving algorithms were designed and refined under real imaging conditions; and the system was evaluated in pool accuracy tests, on dynamic lake data, and in closed-loop lake tracking. These results were obtained at observation distances of 2–6 m under measured 470 nm beam attenuation coefficients of $c=0.5\;m^{-1}$ in the test pool and $c=1.1\;m^{-1}$ in the lake experiments. The collimated-beam attenuation coefficient measured by the C-Star cannot be equated directly with the downwelling diffuse attenuation coefficient used to define Jerlov water types. The operating conditions are therefore reported using the measured attenuation coefficients rather than water-type labels. Future work will extend the evaluation to repeated trials in more strongly attenuating water [54,65], under daytime illumination, at ranges beyond 6 m, and during faster maneuvers. Under these conditions, beam contrast and the random range error, which grows approximately quadratically with distance for a given image-point noise, are expected to become the main limiting factors. It will also address partial beam occlusion and recovery after longer observation gaps, and adapt the marker geometry to other hull sizes, for which the accuracy is expected to depend mainly on the ratio of the marker baseline to the observation distance. Vector-light identity coding for multiple AUVs and joint acoustic–optical tracking will also be investigated.

## Figures and Tables

**Table 1:** Principal processing parameters used in all experiments.

Parameter	Value or Setting
Color-suppression weight α	0.50
CLAHE	Clip limit 1.5; grid 8×8
Segmentation threshold	99th percentile within $M_{v}$
Gaussian filter	5×5 px, σ=1.2 px
Top-hat structuring element	21×21 px
Morphological opening/closing kernel	5×5 px
Hough vote threshold/min. line length/max. gap	50/50 px/25 px
Strip-clustering thresholds $\left(\tau_{\theta },\tau_{\rho },\tau_{g}\right)$	$\left(18^{\circ },5\;\mathrm{px},80\;\mathrm{px}\right)$
Minimum cumulative principal-beam length	120 px
Geometric gate	30-frame window, median ± 3 MAD
TCS weights $\left(w_{e},w_{R},w_{t}\right)$	$\left(1.0\;{\mathrm{px}}^{-1},0.5\;{\mathrm{rad}}^{-1},0.1\;{\mathrm{cm}}^{-1}\right)$
TCS observation-consistency bandwidth $\delta_{e}$	1 px
History reset condition	No valid observation for 2 consecutive frames
LM termination criteria	Maximum 30 iterations; relative cost reduction below $10^{-6}$

CLAHE, contrast-limited adaptive histogram equalization; MAD, median absolute deviation; TCS, temporally continuous candidate selection; LM, Levenberg–Marquardt.

**Table 2:** Principal-beam detection by different line-extraction methods on the dynamic underwater front-end evaluation set (2000 frames).

Method	Successful Detections (Frames)	Success Rate (%)
Standard Hough transform	1827	91.35
Sequential RANSAC line fitting	1453	72.65
LSD segment detection	854	42.70
Hough segments without strip clustering	665	33.25
Proposed Hough–strip method	1997	99.85

All methods take the same enhanced binary beam map as input and differ only in how the four principal beams are recovered from it; the intersections are computed pairwise from the four lines. A detection is counted as successful if all four intersections are obtained and the four fitted lines coincide with the visible beams, as verified manually frame by frame. RANSAC, random sample consensus; LSD, line segment detector.

**Table 3:** Root-mean-square error (RMSE) of each error component of the proposed method in the fixed-pose tests at observation distances of 2, 4, and 6 m.

Error Component (RMSE)		Observation Distance		
		2 m	4 m	6 m
Translation	$e_{x}$ (lateral, cm)	1.0	1.9	2.9
	$e_{y}$ (vertical, cm)	1.1	2.1	3.2
	$e_{z}$ (optical axis, cm)	4.8	10.4	16.8
	$e_{z}/Z$ (%)	2.4	2.6	2.8
Attitude	roll (°)	0.5	1.0	1.6
	yaw (°)	1.2	2.2	3.4
	pitch (°)	2.3	4.2	6.0

All frames were processed with the complete proposed method, as in closed-loop operation. Each distance contains 1000 frames. $e_{z}/Z$ is the depth RMSE normalized by the reference range *Z*.

**Table 4:** Comparison with representative perspective-*n*-point (PnP) solvers and ablation of the proposed solver on an independent continuous dynamic sequence of approximately 2000 frames. Independent ground truth was not available for this open-water sequence; the reprojection error measures agreement with the current observation, and the jitter and jump-rate metrics measure temporal consistency. Trajectory jitter is measured by the root mean square (RMS) of the third-order pose difference over consecutive frames. Translation and attitude jumps use fixed thresholds of 5 cm and 5°, respectively.

Method	Valid Solutions (%)	Reprojection Error (px)		Jitter (Third-Order-Difference RMS)		Jump Rate (%)	
		Median	$p_{95}$	Translation (cm)	Attitude (°)	Translation	Attitude
(a) Representative PnP solvers							
EPnP [48]	83.7	90.42	98.34	20.1	42.9 ^a^	30.66	59.71
IPPE [24]	100.0	3.48	3.81	9.4	311.4	10.07	13.09
SQPnP [49]	100.0	3.47	3.79	9.4	311.4	12.21	6.62
Iterative PnP [23]	100.0	3.47	3.80	9.5	153.7	10.07	12.57
(b) Proposed solver and ablation							
Single initialization (homography–LM)	100.0	3.46	3.81	9.5	139.9	10.07	12.57
Multiple initializations, min. $e_{\mathrm{rep}}$ selection ^b^	100.0	3.45	3.78	9.5	146.2	10.07	12.31
**Proposed**	**100.0**	**3.46**	**3.80**	9.5	**12.6**	**0.00**	**0.00**

^a^ Given the large reprojection errors of EPnP, its comparatively low attitude-jitter value should not be interpreted as evidence of superior estimation performance. ^b^ The same candidate-generation procedure as in the proposed solver (homography-, IPPE-, and previous-pose-initialized LM refinement), with selection by the minimum reprojection error. Bold values indicate the proposed solver. IPPE, infinitesimal plane-based pose estimation; LM, Levenberg–Marquardt.

**Table 5:** Sensitivity of the proposed solver to the TCS parameters on the dynamic sequence. Each parameter was halved or doubled while the others were kept at their nominal values ($\delta_{e}=1$ px, $w_{R}=0.5\;{\mathrm{rad}}^{-1}$, $w_{t}=0.1\;{\mathrm{cm}}^{-1}$).

Setting	Attitude Jump Rate (%)	$p_{95}$ Reprojection Error (px)
Nominal	0.00	3.80
$\delta_{e}=0.5$ px	0.00	3.80
$\delta_{e}=2$ px	0.00	3.80
$w_{R}\times 0.5$	0.00	3.80
$w_{R}\times 2$	0.00	3.80
$w_{t}\times 0.5$	0.00	3.80
$w_{t}\times 2$	0.00	3.80

**Table 6:** Visual-feedback continuity over the lake tracking segment (approximately 214 s).

Statistic	Value
Segment length/frame rate	1071 frames/5 Hz
Duration	214.2 s
Successful feature detection	1069 frames (99.81%)
Passed geometric gate	1066 frames (99.53%)
Valid poses from the proposed method	1065 frames (99.44%)
Longest consecutive loss	1 frame

## Data Availability

The data presented in this study are available from the corresponding authors upon reasonable request. The data are not publicly available because they were collected during field trials subject to institutional restrictions.

## Abbreviations

The following abbreviations are used in this manuscript:

AUV	Autonomous underwater vehicle
CLAHE	Contrast-limited adaptive histogram equalization
DoF	Degree of freedom
IPPE	Infinitesimal plane-based pose estimation
LM	Levenberg–Marquardt
MAD	Median absolute deviation
PID	Proportional–integral–derivative
PnP	Perspective-*n*-point
RMS	Root mean square
RMSE	Root-mean-square error
TCS	Temporally continuous candidate selection
